# Landscape of antitumor CD4^+^ T cells in melanoma:unraveling novel HLA class II-driven immune escape

**DOI:** 10.1038/s41392-022-01307-1

**Published:** 2023-01-16

**Authors:** Xiangjie Xue, Ji Wang, Chenyang Ye

**Affiliations:** 1 Center for Plastic & Reconstructive Surgery, Department of Plastic & Reconstructive Surgery, Zhejiang Provincial People’s Hospital, Affiliated People’s Hospital, Hangzhou Medical College, 310014 Hangzhou, Zhejiang China; 2 Department of Medical Oncology (Key Laboratory of Cancer Prevention and Intervention, China National Ministry of Education, Key Laboratory of Molecular Biology in Medical Sciences), The Second Affiliated Hospital, Zhejiang University School of Medicine, 310009 Hangzhou, Zhejiang China; 3 Cancer Center of Zhejiang University, 310058 Hangzhou, Zhejiang China

**Keywords:** Skin cancer, Tumour immunology

In a recently published article in Nature, Oliveira et al. presented new insights on the relationship between the phenotype and antigen specificity of CD4^+^ tumor-infiltrating cells (TILs) in melanoma, indicating that exhausted cytotoxic CD4^+^ T cells could be directly elicited by HLA class II-restricted neoantigens.^1^

CD4^+^ T cells, as a major type of T cells, play crucial roles in the regulation of tumor immunity. After a certain stimulation, CD4^+^ T cells undergo clonal expansion and differentiate into specific T cell subsets, such as T regulatory cells (Treg), T helper cells, which are closely associated with antitumor responses.^2,3^ Previous study used a combinatorial approach to characterize the T cell receptor (TCR) profile of intratumoral Tregs from patients with metastatic melanoma, which not only revealed the tumor reactivity and neoantigen specificity of Treg-derived TCRs, but also implied that such TCRs could be used for potential antitumor therapeutic approaches.^3^ However, how tumor-infiltrating CD4^+^ T cells are activated to mediate antitumor responses remains unclear. In this paper, Oliveira et al. investigated the phenotype and tumor responsiveness of CD4^+^ T cells derived from melanoma specimens. Their results not only linked the phenotype of CD4^+^ TILs to clonotypes, but also unveiled that tumor-specific CD4^+^ Treg clonotypes could be directly stimulated by HLA class II positive melanoma in the presence of extremely high tumor mutation burden (TMB).

To determine the relationship between different expression patterns of melanomas and the TCR clonotypes of CD4^+^ TILs, Oliveira et al. collected 5 tumor specimens from 4 melanoma patients and obtained scRNA-seq data from the isolated cells. Uniform manifold approximation and projection (UMAP) was performed to classify CD4^+^ TILs into 10 clusters. Although most TCRs were distributed in different clusters, the results showed that most T cells carrying the same TCRs were restricted to clusters with one particular pattern, namely: the predominant acquisition of an exhausted phenotype (TEx). CD4^+^ TILs in TEx included the following three clusters: Terminally exhausted (TTE) TILs, Follicular helper with features of progenitor exhausted (TFH/TPE) TILs and Proliferating (TProl). Also, significant clonotypic amplification of CD4^+^ Treg TILs was observed in HLA class II positive melanoma, while no significant amplification was observed in other types of TILs. Their data indicated that highly amplified clonotypes were mainly distributed in TEx, and partially in Treg TILs, implying that highly amplified clonotypes play pivotal roles in antitumor responses.

To further illustrate the relationship between TCRs and tumor recognition, Oliveira and colleagues evaluated the responsiveness and specificity of different types of TCRs to tumors in light of CD137 upregulation, revealing that TTE TCRs were highly enriched for antitumor specificity. Also, HLA class II positive melanoma directly participates in and stimulates TCR expressed by CD4^+^ Treg, while in the absence of HLA class II tumor expression, TILs can be stimulated indirectly by stimulating tumor antigens through Antigen-presenting cells (APCs). This finding further clarified the relationship between tumor-specific CD4^+^ T cells and melanoma in tumor microenvironment (TME). Co-cultured with peptide-pulsed monoallelic HLA class I-expressing targets, TCR-transduced CD4^+^ effectors recognized their cognate antigens within HLA class I restriction, suggesting that the HLA class II positive melanoma TME is rich in neoantigen-specific Treg cells. Also, the results revealed the responsiveness of different TCRs to melanoma and the impact of antigen specificity on tumor recognition in HLA class II positive and negative melanoma. Together, Oliveira et al. identified that neoantigen and HLA class II expression had favorable effects on orchestrating interactions with immunosuppressive cells in TME.

Tumor mutational burden (TMB) reflects cancer mutation quantity. Mutations are processed to neoantigens and presented by major histocompatibility complex (MHC) proteins to T cells including melanoma.^4^ The association between HLA class II expression and TMB was analyzed using 116 melanoma specimens from 4 cohorts (DFCI-Neovax18, MGH3, Checkmate 064, Checkmate 038), indicating that 28 HLA class II positive melanomas had higher TMB than 87 HLA class II negative melanomas. The HLA class II upregulation was enriched in tumors with extremely high TMB. High TMB associated with HLA class II positive melanoma and the ability of neoantigens generating CD4^+^ and CD8^+^ TILs are the two major factors leading to the results.

Oliveira et al. investigated on the phenotype and clonotype of infiltrating CD4^+^ T cells in melanoma, illustrating how tumor-specific CD4^+^ TILs with different HLA expressions could engage with tumor cells to induce antitumor responses in TME. In addition, the TME of HLA class II positive melanoma is enriched with neoantigen-specific Treg. However, HLA class I-restricted CD4^+^ TILs have not been thoroughly explored, and further studies are needed to explore their role in antitumor responses. Oliveira et al. also found that HLA class II positive melanomas were characterized not only by clonal expansion of Treg cells, but also by high numbers of CD8^+^ TILs, owing to their association with extreme TMB.^1,5^ The recovery of cytotoxic CD8^+^ responses may disrupt the delicate equilibrium between Treg and effector cells, leading to enhanced immunogenicity of HLA class II positive melanoma, which further provides a plausible immunological rationale for improving the immunotherapy of melanoma. Recently, targeting Tregs to improve efficacy of cancer immunotherapy is being intensively studied. It would be interesting if specific depletion or function suppression of immunosuppressive CD4^+^ TReg cells is feasible in future research. In addition, whether the cellular characteristics of CD8^+^ TILs modulate the clonal expansion of CD4^+^ TILs remains to be explored. And the potential interaction between CD8^+^ and CD4^+^ TILs is also worth noting, and requires further investigation.

**Fig. 1:**
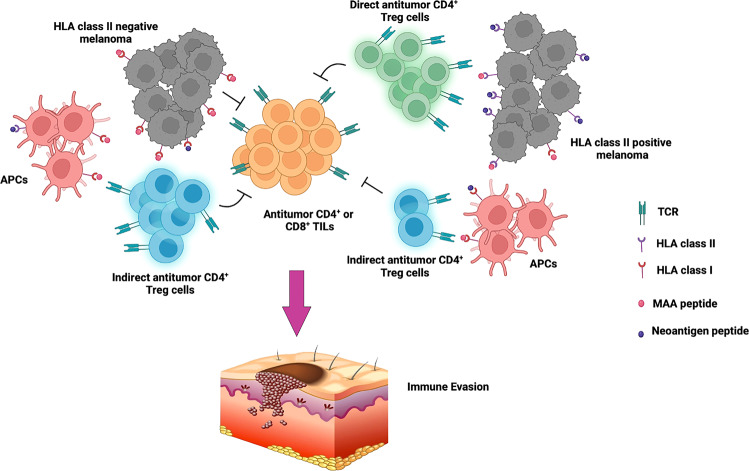
Novel mechanism of melanoma HLA class II-driven immune escape. With the assistance of indirect antitumor CD4^+^ Treg cells presented by Antigen-presenting cells (APCs), and direct antitumor Treg cells (major tumor-reactive cells), HLA class II positive melanoma cells evade immune surveillance. In addition, Melanoma directly induced cytotoxic CD4^+^ T cells depletion through HLA class I, but not directly induced Treg production through HLA class I. The figure was generated on Biorender.com

Taken together, the study by Oliveira and colleagues unveils the in-depth characterization of treatment-naive melanomas, disclosing the relationship between tumor-specific CD4^+^ TILs and tumor cells (Figure 1). Their findings proved that de novo antigens of melanoma mediate immune evasion mainly through HLA-II induction of local negative immune regulatory CD4^+^Treg cells. Undoubtedly, their intriguing results provide new insights to better understanding of immune escape mechanism at single-cell resolution, and inspire personalized treatment such as immune checkpoint therapy and cancer vaccines for better clinical efficacy. Fig. 1
